# Regulation of key proteins in Alzheimer’s disease molecular pathogenesis by ubiquilin-1

**DOI:** 10.1186/1750-1326-8-S1-P20

**Published:** 2013-09-13

**Authors:** Mari Takalo, Jayashree Viswanathan, Teemu Natunen, Kaisa MA Kurkinen, Heikki Tanila, Hilkka Soininen, Rudolph E Tanzi, Mikko Hiltunen, Annakaisa Haapasalo

**Affiliations:** 1 Institute of Clinical Medicine – Neurology, University of Eastern Finland and Department of Neurology, Kuopio University Hospital, Kuopio, Finland; 2 Genetics and Aging Research Unit, Massachusetts General Hospital and Harvard Medical School, Charlestown, MA, USA

## Background

Ubiquilin-1 is a ubiquitin-like protein involved in the pathogenesis of Alzheimer’s disease (AD) and other neurodegenerative disorders via different mechanisms. *UBQLN1 UBQ-8i* genetic variation associates with increased AD risk. Ubiquilin-1 protein functions as a molecular chaperone controlling amyloid precursor protein (APP) trafficking and processing and presenilin (PS) levels and subcellular targeting. Ubiquilin-1 harbors a ubiquitin-like (UBL) domain interacting with the proteasome and a ubiquitin-associated (UBA) domain binding polyubiquitinated proteins. Thus, it may target polyubiquitinated proteins to degradation. Alternative splicing of *UBQLN1* generates four transcript variants (TV) with varying domain structures, suggesting that diverse variants may specifically interact with and regulate different proteins and cellular functions.

## Materials and methods

HEK293 or SH-SY5Y cells overexpressing APP751 were transfected with ubiquilin-1 TVs containing myc and/ or mRFP tags and PS1 or 0N4R-tau using Lipofectamine^®^. Cultured E18 embryonic cortical neurons from transgenic APP/PS1 mice were transfected using magnetofection or lentiviruses. Proteins of interest were assessed from protein lysates, membrane fractions, or immunoprecipitates by Western blotting. Intracellular localization of proteins was examined by confocal microscopy.

## Results

We found that TV1, the full-length ubiquilin-1, and TV3, containing an incomplete UBL domain, caused accumulation of high-molecular-weight (HMW) PS1 and targeted accumulated PS1 to intracellular inclusions termed aggresomes. TV3 induced PS1 accumulation and aggresomal targeting more strongly than TV1, suggesting that TV3 may not target PS1 to proteasomal degradation as efficiently as TV1. In addition, we discovered that TV1 increased the ε-site cleavage of APP and another PS/γ-secretase substrate, resulting in increased release of the respective intracellular domains but no overt changes in Aβ levels. Interestingly, TV2, whose levels increase in AD brain by the *UBQ-8i* variation, did not have a similar effect. We next investigated whether ubiquilin-1 affects the levels of tau, another key protein in AD and other tauopathies. We observed that TV1 increased the levels and phosphorylation status of tau. However, we found no evidence for HMW-tau and tau did not localize in intracellular inclusions similarly to PS1. Further induction of protein accumulation by proteasomal inhibition did not result in the formation of tau-containing inclusions nor did it potentiate the increase in tau levels by TV1. We are currently investigating the mechanisms by which ubiquilin-1 regulates the AD-related proteins and the potential subsequent implications in AD molecular pathogenesis *in vitro* and *in vivo.*

## Conclusions

Our data suggest that ubiquilin-1 functions as a modulator of PS/γ-secretase cleavage and as a molecular chaperone for APR, PS1 and possibly tau. These findings agree well with the previously suggested function for ubiquilin-1 as a regulator of protein levels, degradation, and targeting to inclusions. Our results further propose that different ubiquilin-1 TVs may have differential functions, which may have important implications in the pathogenesis of AD and other neurodegenerative disorders.

